# Management of scientific and ancestral knowledge: a decision-making model in mezcal industry in Mexico

**DOI:** 10.3389/frai.2025.1570617

**Published:** 2025-05-21

**Authors:** Antonia Terán-Bustamante, Antonieta Martínez-Velasco, Sandra Nelly Leyva-Hernández

**Affiliations:** ^1^Universidad Panamericana, Facultad de Ciencias Económicas y Empresariales, Ciudad de México, Mexico; ^2^Facultad de Ingeniería, Universidad Panamericana, Ciudad de México, Mexico; ^3^Tecnológico Nacional de México, Instituto Tecnológico del Valle de Etla, Oaxaca, Mexico

**Keywords:** scientific and ancestral knowledge, indigenous communities, decision-making model, sustainability, Mexico, fuzzy logic model, machine learning, low-tech

## Abstract

**Introduction:**

Knowledge management is essential to ensure the sustainability of rural communities and small producers since it generates value for innovation, productivity, and competitiveness. The aim of this study is to identify relevant factors for adequate decision-making in managing knowledge in the Mexican mezcal industry and its impact on developing rural communities and small producers - mezcaleros. For this purpose, a decision-making model for managing scientific and ancestral knowledge is created to support links with universities, research centers, and rural communities to accelerate innovation and competitiveness in this sector.

**Methods:**

The analysis methods were carried out through decision-making, machine-learning techniques, and fuzzy logic.

**Results:**

The Bayesian Network model suggests that the preceding variables to optimize the Mezcaleros Knowledge Management are the Mezcaleros Indigenous community, the Denomination of Origin, Scientific and Ancestral Knowledge, Waste Management and Use, and *Jima*.

**Discussion:**

This knowledge management model aims to guide small producers to be more productive and competitive through the support of a facilitator.

## 1 Introduction

*Mezcal*, an emblematic agave-distilled drink from Mexico, is steeped in a rich cultural heritage. It has earned recognition as a quality product on national and international scales, underscored by its prestigious Denomination of Origin. This legal term signifies the product’s unique geographical origin and quality (Álvarez Hernández and Mercado Salgado, 2022). Mezcal production is not merely a process but a cornerstone of our cultural identity and sustainability. It symbolizes the profound connection between communities and their ancestors, bridging our past and future. The production and consumption of this beverage are deeply intertwined with celebrations, ceremonies, and the cultural identity of communities, making it an integral part of their lives (Álvarez Hernández and Mercado Salgado, 2022; Ávila-Reyes et al., 2025).

The mezcal industry, intricately linked to agave agriculture, faces many challenges that imperil its sustainability and growth. While many communities adhere to traditional farming practices that are not only environmentally friendly but also promote biodiversity (García and González, 2021), the industry is grappling with significant obstacles that demand urgent attention and intervention.

One of the most pressing issues is the inadequate infrastructure in many producing communities. This lack of access to modern technology, essential services, and resources limits the capacity of these communities to scale up production efficiently. As a result, many small producers cannot compete in a rapidly evolving market (Castillejo-Reyes et al., 2023).

Additionally, the mezcal industry relies on a limited number of agave species for production—approximately nine species are utilized. However, troublingly, only one of these is actively nurtured for future reproduction. The remaining eight species exist in the wild, making their collection unsustainable and leading to a significant decline in populations of crucial varieties, such as Tobalá maguey (Agave potatorum), which is vital for mezcal manufacture (Rodríguez-Peralta et al., 2019). This scenario results in the overexploitation of agave species to satisfy increasing global demand (Arellano-Plaza et al., 2022). The consequences of overharvesting are compounded by the reproductive characteristics of agave, which reproduces exclusively through seeds. This singular method of propagation has led to alarming reductions in agave populations (Castillejo-Reyes et al., 2023), further endangering the long-term viability of the mezcal industry. Sustainable seedling production schemes are necessary (Cervantes-Luna et al., 2022).

Phytosanitary challenges also plague mezcal production areas. Producers often face issues such as plant diseases and infestations, alongside other factors that jeopardize the health and productivity of agave plants. These problems can severely impact both the quality and quantity of mezcal produced.

Economic factors exacerbate these challenges, as producers often receive low compensation for their raw materials. As input costs, particularly for packaging, continue to rise, profitability remains elusive for many in the industry.

Despite the involvement of various research groups in improving agave cultivation, the connection between these organizations and small producers often remains tenuous. The lack of effective communication and knowledge transfer hampers the potential for innovation and improvement, highlighting a key issue in the industry. Academic institutions and research centers often overlook and undervalue the traditional knowledge and practices passed down through generations within local communities. However, recognizing and integrating this wealth of traditional knowledge with current scientific research could play a pivotal role in addressing the challenges the mezcal industry faces, offering a potential avenue for positive change.

Currently, knowledge management is not just a fundamental element of sustainability; it is the key to our future. Through it, organizations generate value. Therefore, managing it to obtain information and disseminating it systematically and efficiently to transform it into helpful knowledge that can be quickly included in decision-making and strategies represents a competitive advantage to generate innovative actions (Terán-Bustamante et al., 2021, 2022, 2025; Martínez-Velasco et al., 2024; Flores Torres et al., 2021).

In this context, the management of scientific and ancestral knowledge in the mezcal industry in Mexico is essential to promoting sustainability, quality, and cultural heritage. That is, improving the quality and sustainability of the product, people, and the entire ecosystem to strengthen the culture, regional identity, and the economic viability of producing communities. Therefore, this research aims to create a model that allows decision-makers and public policymakers to focus on policies that recognize and support ancestral and scientific knowledge, which can help producers access resources, finance, and training and promote industry development.

However, few studies combine the study of traditional methods with scientific techniques focused on improving the quality of mezcal. These techniques maintain the product’s authenticity while optimizing processes such as fermentation and distillation. At the same time, they have a social impact that provides a better quality of life for Indigenous and rural communities. Emerging from the previous situation, the questions that drive the research are: What are the essential factors for optimal decision-making for scientific and ancestral knowledge management in the mezcal sector in Mexico? How can small producers in the mezcal sector, using a decision-making ancestral knowledge management model, make better decisions to innovate new products, services, processes, and business models that allow them to be more competitive? What are the best correlations between the factors in the mezcal sector’s scientific and ancestral knowledge and innovation management model to generate value for small producers?

This work is structured in three sections. The first section deals with the theoretical framework, specifically the conceptualization and matter of scientific and ancestral knowledge management. It also characterizations the mezcal sector in Mexico and its production process. The second section presents the methodology, the construction of the conceptual model based on Bayesian Networks (BNs), and analysis with machine learning and fuzzy logic. Finally, the third section presents the results, discussion, and conclusions.

## 2 Theoretical framework

The increasingly evident complexity of human reality and its relationship with the environment, with others, and with the whole highlights, on the one hand, the opportunity to acquire a phenomenological practice to increase awareness and, on the other hand, the urgency of interweaving the multiple fields and research perspectives developed (Colonna, 2024), for example, this is the case of scientific knowledge of the ancestors. According to Colonna (2024), scientific knowledge is beginning to break down established disciplinary barriers, and the search for ways to integrate worldviews is returning to an approach to the commons of shared knowledge of humanity.

### 2.1 Management knowledge and scientific and ancestral knowledge in the mezcal production process

According to Dalkir (2011), knowledge is information that resides in people’s minds and is used to make decisions or take actions in unfamiliar contexts, generating value.

Therefore, organizations alone cannot create knowledge; they make it with people, with their talent (Terán-Bustamante et al., 2021). For this knowledge to generate this knowledge, it must be shared with other people, disseminated, and amplified at the group level to form a spiral that, through different ontological levels, becomes one of the keys to its creation (Terán-Bustamante et al., 2021). That is, it is necessary to understand how something works, and it fundamentally involves interrelationships and behavior. Therefore, knowledge is dynamic, created through social interactions between individuals and organizations (Fearnley and Horder, 1997).

Therefore, knowledge management is a key element that allows capturing the collective experience of an organization to make it more innovative, productive, and competitive (Dalkir, 2011; Terán-Bustamante et al., 2021).

Although ancestral knowledge and scientific have different origins and approaches, they can complement each other in resource management and problem-solving. Ancestral knowledge, based on practices and wisdom passed down from generation to generation, provides a view of nature and the world from the perspective of local culture, while scientific knowledge uses methods to understand and explain natural phenomena (Pinedo, 2021; Echavarría-Heras et al., 2023).

Therefore, in the case of the integration of ancestral knowledge and scientific knowledge, both forms of knowledge can lead to more sustainable and effective resource management, as well as to the identification of innovative solutions to environmental and social problems (Pinedo, 2021; Echavarría-Heras et al., 2023).

Traditional knowledge is rooted in the experiences of individuals and is passed down through generations via storytelling and oral traditions. This knowledge is vital for many communities as it helps them address the challenges they face in their daily lives (Briones et al., 2021). By combining traditional and academic knowledge, they can tackle issues that hinder their development and improve their quality of life (World Wildlife Fund Inc., 2023). According to Nonaka and Teece (2001), as well as Teece (2023) and Terán-Bustamante et al. (2021, 2022), knowledge is context-dependent and influenced by people; it involves understanding how things work and recognizes interrelationships and behaviors (Martínez-Velasco et al., 2024). Knowledge management is, therefore, about ensuring that the proper knowledge reaches the right people at the right time.

In the production of mezcal in Oaxaca, ancestral knowledge plays a crucial role, as mezcal artisans acquire their expertise from their parents or family members (Consejo Mexicano Regulador de la Calidad del Mezcal, 2022; Sandoval-Aragón, 2020). This ancestral knowledge is transmitted in various forms, from simple conversations between family members to more complex expressions, including narratives, dances, ceremonies, and rites, mainly as reflected in mezcal festivals (Briones et al., 2021; Benites, 2025).

Rural communities have coexisted with nature, relying on it for their livelihoods. This close relationship fosters a sense of stewardship and interdependence, allowing them to generate knowledge based on personal experience. Recognizing patterns, processes, and relationships within their environment is unique to these communities and empowers them to adopt sustainable practices (World Wildlife Fund Inc., 2023). Effective management of ecosystems and natural resources necessitates a comprehensive understanding of complex socio-ecological dynamics, including their use, conservation, restoration, and management across different geographical scales (Vázquez-Pérez et al., 2020; Lira et al., 2022a, 2022b). The sustainable management of mezcal production is essential for a thorough analysis of knowledge management (Contreras-Medina et al., 2021).

Various ecological practices are integrated into the intricate mezcal production process to promote sustainability and environmental stewardship. A sustainable process requires adopting sustainable energy sources, reducing and reusing resources, and reducing the exploitation of wild agave (Jaramillo-Villanueva and García-Benítez, 2024). One notable method involves the innovative use of agave residues in cooking ovens, which serve not only as an energy source but are also repurposed as an organic substrate for greenhouses and as a natural fertilizer for crop fields (Martínez-Velasco and Terán-Bustamante, 2022). Additionally, promoting reforestation initiatives using indigenous plant species enhances biodiversity, while water recirculation in the production process minimizes resource waste. The establishment and nurturing of nurseries play a crucial role in ensuring a steady supply of agave plants and fostering genetic diversity within these crops (Ruiz Mondragón et al., 2023).

In managing agave plantations, employing a rotational and associative cultivation approach with staple crops like corn, beans, and squash—often seen in the traditional milpa agroecosystem—creates an environment conducive to cultivating agaves with heightened genetic variability. This collaborative farming method bolsters food security for the producing communities and preserves essential plant varieties for future generations (Cobos et al., 2023). The symbiotic relationship between the milpa system and cultivating various local agave species for mezcal production increases land productivity and encourages rainwater’s natural retention and filtration. This dynamic, in turn, mitigates soil erosion and reduces pest infestations, contrasting sharply with the unsustainable monoculture practices prevalent in tequila production (de la Torre and Gordon, 2018).

At the heart of these efforts lies a profound connection between local communities and their natural surroundings. The perception of this relationship significantly influences conservation practices and highlights the invaluable role of rural populations in safeguarding agrobiodiversity—a unique and irreplaceable contribution (Agricultura Sostenible, 2021). According to Loyola (2016), integrating ancestral and traditional knowledge into rural family agricultural systems is crucial for fostering sustainable practices, ensuring that these communities’ ecological and cultural heritage thrives amid modern challenges.

Family farming is an agricultural and social activity that involves cultivation and production for internal consumption and surpluses for marketing. Family members participate in both work and decision-making. Family farming is based on cultural procedures at the farm, locality, or territory level, preserving traditions and knowledge about food production and ecological practices that respect nature (Loyola, 2016). However, these virtues of family farming are often not recognized, thereby marginalizing the potential to contribute to any country’s broader social development. However, only the interaction between family members can represent an imbalance in socialization for knowledge management since interaction with external agents is almost nonexistent (Flores Torres et al., 2021). Therefore, it is crucial to understand knowledge management in the mezcal production process to ensure its sustainability.

**Figure 1:**
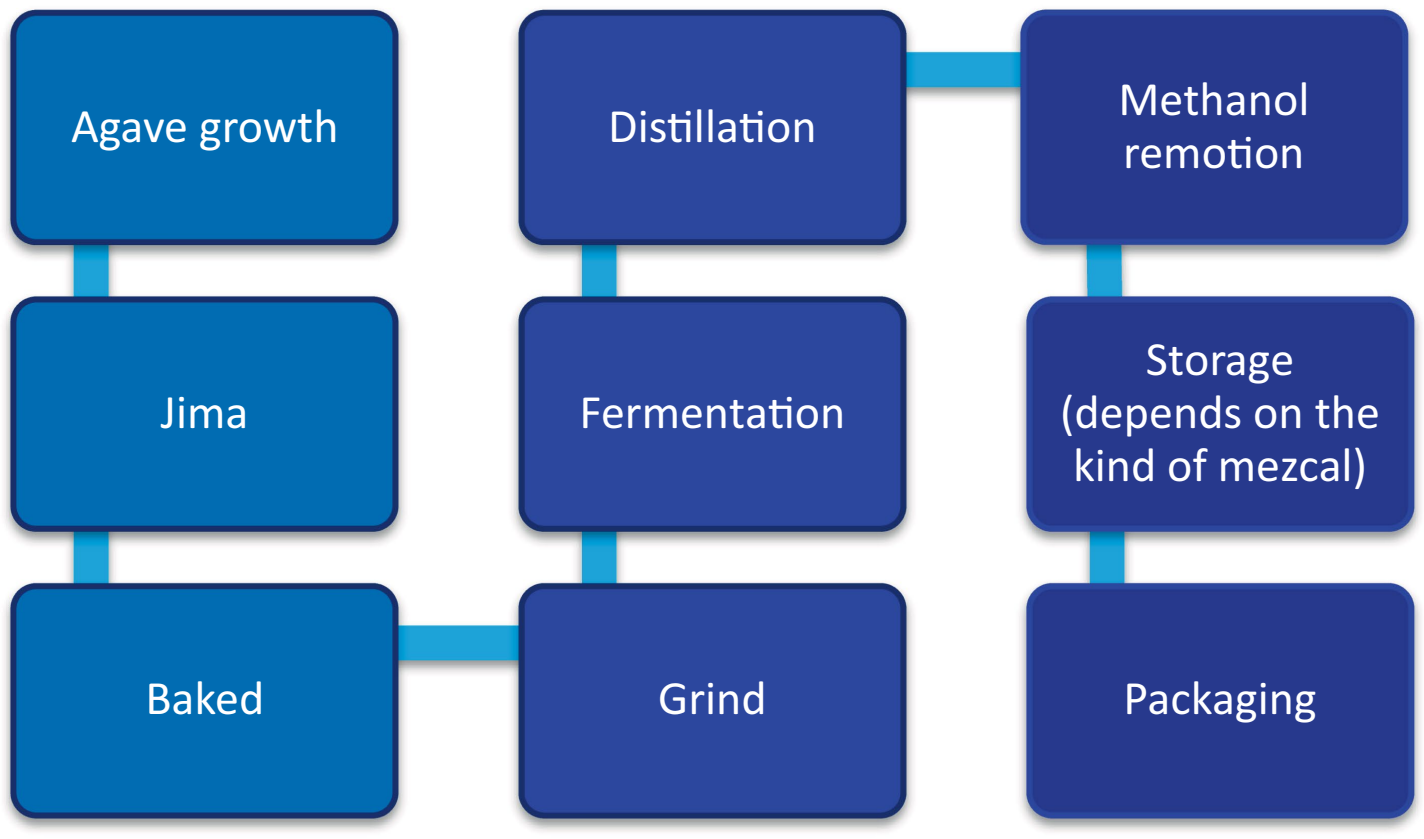
Mezcal’s manufacturing process. Source: own elaboration based on Contreras-Medina et al. (2021), Sandoval-Aragón (2020), García et al. (2024), and Norma Oficial Mexicana NOM-070-SCFI-2016, Bebidas Alcohólicas-Mezcal-Specificaciones (2016).

Mezcal production is a complex process that includes 8–9 stages, from the agave to the mezcal’s maturation, depending on the type of mezcal (Arellano-Plaza et al., 2022; Ávila-Reyes et al., 2025). The stages of mezcal production are agave maturation, jima, cooking, grinding, fermentation, distillation, refining, packaging, and maturation of mezcal (Contreras-Medina et al., 2021; Sandoval-Aragón, 2020; García et al., 2024) (Figure 1).

The production of mezcal involves the incorporation of wild agaves collected in the surroundings of the producing communities (Arellano-Plaza et al., 2022). Consumers prefer Wild agaves (World Wildlife Fund Inc., 2023), such as Agave potatorum, with which Tobalá mezcal is produced, and it is appreciated by consumers and producers for its flavor (Ruiz Mondragón et al., 2023). However, due to the demand for this agave, its population has decreased since growers do not expect the species to produce flowers (Agricultura Sostenible, 2021). This decline in agave species could disrupt the mezcal industry, highlighting the urgent need for sustainable agave cultivation practices. The pollination of agaves depends on bats and other animals (Ruiz Mondragón et al., 2023). As a solution to the loss of this species in the wild and the supply of mezcal production, its cultivation in greenhouses has been proposed (World Wildlife Fund Inc., 2023). It is considered a critical factor for the sustainability of mezcal businesses, participation in the reproduction of agaves in backyard or community greenhouses, and seed collection (Barrientos-Rivera et al., 2020).

The Denomination of origin aims to validate the artisanal process and traditional knowledge through a distinctive mark (Domínguez-Arista, 2020). This mark protects the production of drinks and agave within a specific territory (Arellano-Plaza et al., 2022). However, this practice can have the opposite effect on the management of artisanal knowledge. Sometimes, preserving this knowledge through standardization processes can disconnect it from the cultural heritage, including the producing communities’ practices, beliefs, and worldviews (Lira et al., 2022a, 2022b). Therefore, it is crucial to understand how it is connected to other elements of knowledge management in this sector.

Certification, branding, and formalization of products present significant challenges for producers, especially regarding costs and procedures. Access to large, highly demanding markets, such as those in Mexico City and abroad, is typically limited to companies with greater productive capacities. These companies can navigate the industrial production certification processes and buy bulk from small producers. However, such companies are few in the industry (Barrientos-Rivera et al., 2020; Martínez-Velasco and Terán-Bustamante, 2022).

Most artisanal mezcal producers sell their products to intermediaries, who market them to the public. Only a few can sell directly to consumers. Two types of organizations market mezcal: one comprises small producers who sell locally or to intermediaries (Nieto-García and Toledo-López, 2024). In addition to the concentration of marketing in packaging companies, a significant income gap exists between what producers receive and what intermediaries earn. When a mezcal producer sells wholesale, they receive approximately 25 Mexican pesos per liter, while marketers can charge between 180 and 2000 Mexican pesos for 750 milliliters of mezcal (Vera and Akaki, 2017). Due to low prices for their mezcal and payment in installments, producers also face the risk of not receiving their payment (Bautista et al., 2015). This situation contributes to a decrease and abandonment of artisanal production as the industrialization of the drink gains favor, leaving producers vulnerable (Bautista and Melchor, 2008).

Mezcal production is supported by two types of markets: one that requires a certified product and one that does not depend on certification. Both markets require processes of social and political integration. In the context of crafts, social integration holds cultural value as it is part of peasant economies, dialogue of knowledge, and traditional technologies. The product’s sale is conducted directly from producer to consumer, involving cultivation, care, classification, storage, transformation, transportation, and sale based on agroclimatic factors related to the agave’s maturation. The quality of mezcal is assessed not only by alcohol concentration but also by environmental aspects.

Artisanal mezcal production relies on ancestral knowledge about cultivation, harvesting, and distillation passed down through generations. This knowledge helps preserve the histories and cultural legacies of communities. Thus, it is essential to manage ancestral knowledge alongside scientific knowledge, particularly considering the involvement of various actors. When this collaboration and knowledge exchange occurs, all participants, especially the rural and Indigenous communities—known as mezcaleros—can leverage this collective knowledge to enhance efficiency and productivity. Local innovation should contribute to sustainability and improve quality of life (World Wildlife Fund Inc., 2023; Contreras-Medina et al., 2021; Pérez-Salas et al., 2024).

## 3 Materials and methods

**Figure 2:**
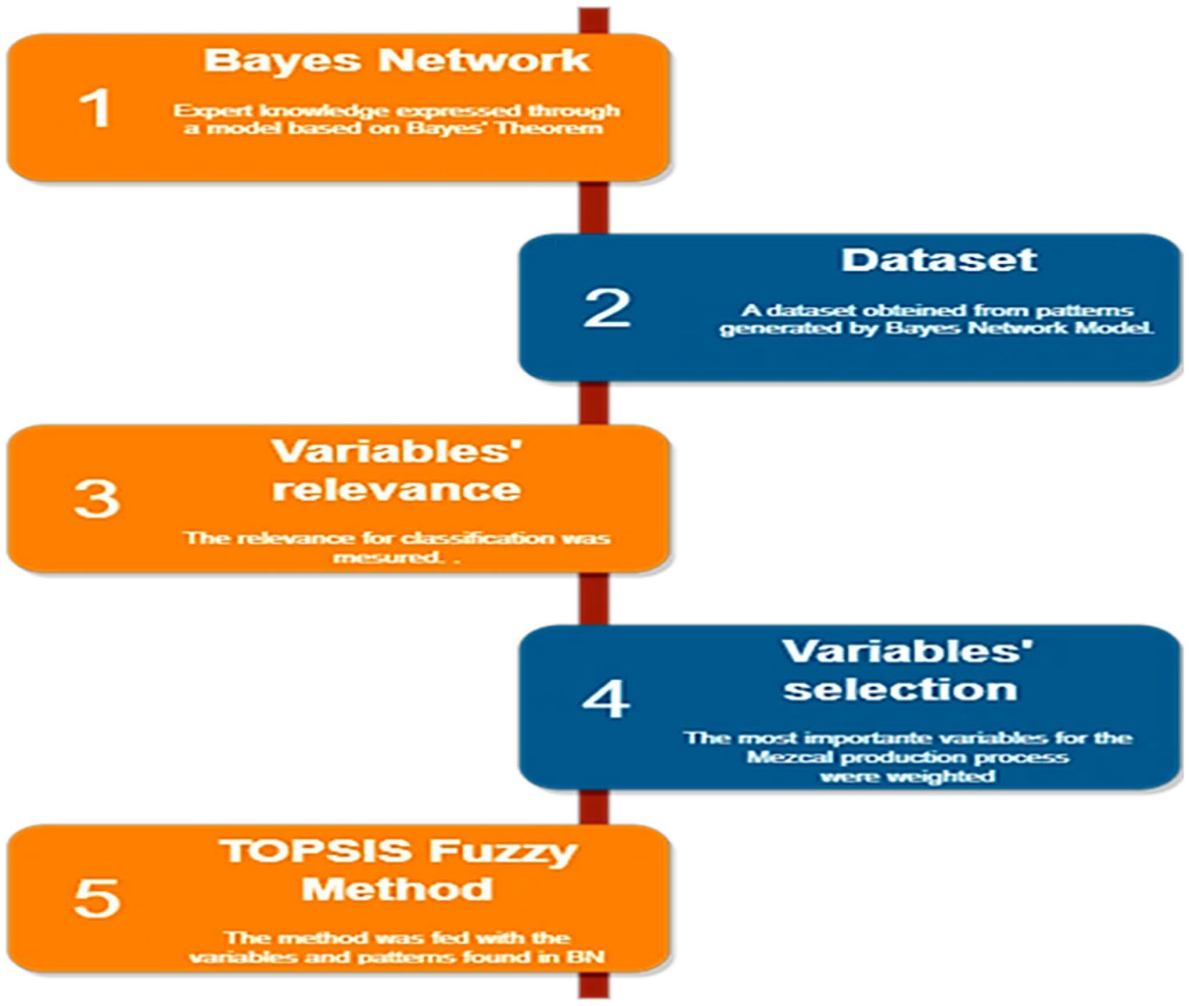
The methodology applied to determine the best alternatives to achieve the optimum level of knowledge management.

Expert insights on Mezcal production served as the foundation to conduct this study. A Bayesian Network (BN) was constructed using the gathered data, forming a decision-making model for integrating scientific and ancestral knowledge. This model illustrates the connections between universities, research centers, and rural communities, aiming to accelerate innovation and enhance competitiveness within the Mezcal industry. Subsequently, a dataset was generated based on the patterns identified in the proposed BN. From this dataset, the most critical variables for classification were determined by applying the Information Gain metric, which measures the amount of information each variable provides (in terms of entropy reduction). After placing the key variables needed to optimize the target variable (Knowledge Management), the Fuzzy TOPSIS method was employed as the final step. This approach identified the best combinations of the selected variables to achieve optimal outcomes in Knowledge Management (Figure 2). The techniques applied in this study is briefly outlined below to clarify the procedure followed.

### 3.1 Bayesian networks

Bayesian networks (BNs) are probabilistic graphs representing a field’s uncertainties. BNs represent the domain variables and their dependencies. The use of BNs stands out in its explicit attitude toward uncertainty, its facileness in estimating the state of specific variables given some evidence, and its support methods for decision analysis and fast user responses.

In probability theory, a domain D and its uncertainties are modeled by a collection of random variables $D=\{X_{1},\ldots ,X_{n}\}$ . Each random variable $X_{i}$ has a cluster of possible values that merge, constructing the basis for the modeling of domain D. The occurrence of each combination is measured using probabilities specified by a joint probability distribution.

A Bayesian network is a Directed Acyclic Graph (DAG) which encodes a joint probability distribution in a set of variables D. in this way, a network is defined by the pair B={G,θ} . where G is a DAG whose vertices correspond to the variables $X_{1},\ldots ,X_{n}$ . The edges represent directed dependencies among variables symbolized by circles, and the borders are illustrated by arrows indicating the causal connection’s direction. The global semantics of BN specifies that the product gives the full joint distribution

$$P(X_{1},\ldots ,X_{n})=\prod_{i}P(X_{i}|{\mathrm{pa}}_{i})$$

The learning problem of BN has two components: the construction of structures and the set of parameters for the DAG. The structure can be built from data or experts “knowledge where no data is available before construction.” Consequently, given this distribution, generating a data file is sometimes helpful in learning from the BN structure. Once the data was generated, it could be analyzed using other techniques to obtain a better combination of variables to achieve a higher value for the target variables. In this way, we processed the dataset, scoring the variables according to the Information Gain Method (IGM) to measure the scores of the variables based on their correlation with the target variable. For categorical variables, IGM assesses the information gain achieved by splitting the data based on the different categories of that variable. Then, Information gain is a measure of the reduction in entropy (uncertainty) of the target variable when a particular feature is known.

The information gain is the reduction in information entropy H from a prior state to a state that takes some information as given:

IG(T,a)=H(T)−H(T∣a)

Where H(T∣a) is the conditional entropy of T given the value of attribute *a*.

Next, the values of the information gain metric are used to determine which variables to consider when integrating them into the developed multi-criteria decision analysis method.

### 3.2 Fuzzy multicriteria decision making problem formulation

The Technique for Order of Preference by Similarity to Ideal Solution (TOPSIS) is a multi-criteria decision analysis method developed by Hwang et al. (1993) and Tzeng and Huang (2011). TOPSIS is based on the idea that the selected alternative should have a brief geometric distance from the positive ideal solution (PIS) and the longest from the negative ideal solution (NIS).

In order to find the best combination among variables, a Multicriteria Decision Making (MCDM) problem by m alternatives {*A*1, *A*2, …, *A*m} which should be calculated by applying n criteria {*C*1, *C*2, …, *C*n} can be bring up by the decision matrix.

$$x=[\begin{array}{cc} x_{11} & x_{12}\;\ldots \;x_{1n}\\ \begin{array}{c} x_{21}\\ \ldots \\ x_{m1} \end{array} & \begin{array}{c} x_{22}\;\ldots \;x_{2n}\\ \begin{array}{ccc} \ldots & \ldots & \ldots \end{array}\\ \begin{array}{ccc} x_{m2} & \ldots & x_{\mathrm{mn}} \end{array} \end{array} \end{array}]$$

Where $x_{\mathrm{ij}}$ is a numeric data which denotes the value of the $i_{\mathrm{th}}\;$ alternative with respect to the $\;j_{\mathrm{th}}$ criterion. The importance (or weight) of the criterion C_j to the decision is denoted by w_j . Let stat w be the vector.

$$w=[\begin{array}{ccc} w_{1}, & w_{2}, & \begin{array}{cc} \ldots , & w_{n} \end{array} \end{array}]$$

Generally, the weights are determined subjective by a single decision-maker or a group of experts. The TOPSIS method can rank alternatives in Multiple-criteria decision-making (MCDM) problems. It has significant advantages, such as quickly organizing the best options and dealing with conflict situations. However, the method has disadvantages; in the classical TOPSIS, numerical values represent the personal judgments of decision-makers. The TOPSIS method could be more efficient in estimating people’s selections with precise data because human appraisals are imprecise and ambiguous. To overcome these deficiencies, the Fuzzy TOPSIS method is suggested to consider the fuzzy values inherent in human opinions (Krohling and Campanharo, 2011).

**Figure 3:**
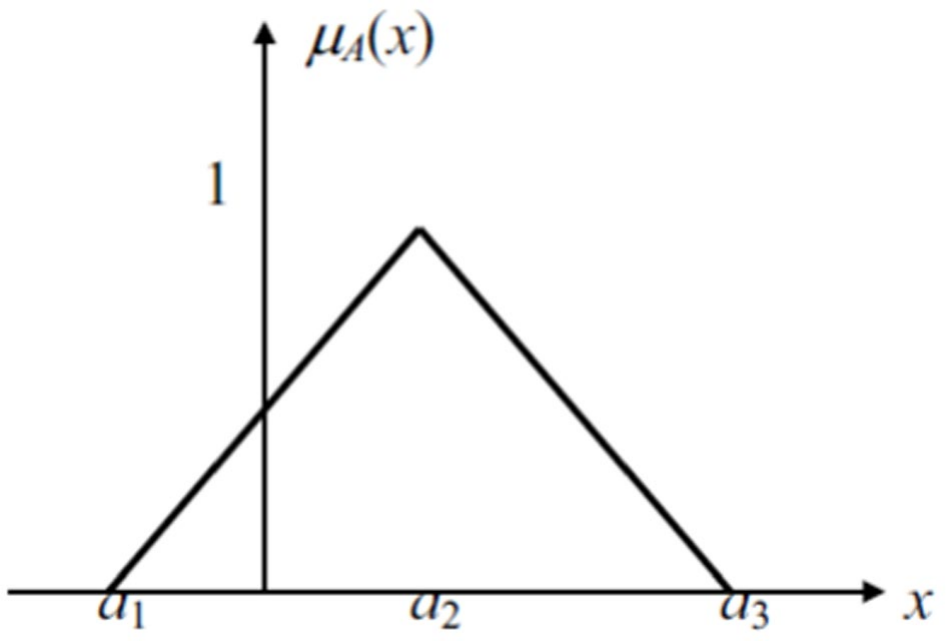
Triangular fuzzy number A = (a1, a2, a3).

Likewise, the Fuzzy TOPSIS method is uncomplicated in solving MCDM problems with imprecise data. In this method, linguistic variables are applied to estimate the weights of all criteria and the ratings of all choices, which are converted into triangular fuzzy numbers (TFNs). A fuzzy number is a generality of a regular actual number. It is not guided to one value but to a connected collection of probable values, where each possible value weighs 0 and 1 (Nădăban et al., 2016). Of the various shapes of fuzzy numbers, triangular fuzzy numbers (TFN) are the most widespread for their plainness and efficiency in the results. Where a Triangular fuzzy number is a fuzzy number denoted by three points as A=(a1,a2,a3) . The representation is understood as membership functions (Figure 3).

Where:

$$\mu_{\left(A\right)}(x)=\{\begin{array}{cc} \begin{array}{c} 0,\\ \frac{x-a_{1}}{a_{2}-a_{1}}\\ \begin{array}{c} \frac{a_{3}-x}{a_{3}-a_{2}}\\ 0, \end{array} \end{array} & \begin{array}{c} x<a_{1}\\ a_{1}\leq x\leq a_{2}\\ \begin{array}{c} a_{2}\leq x\leq a_{3}\\ x>a_{3} \end{array} \end{array} \end{array}$$

If a crisp interval is obtained by *α*-ration, interval Aa could be brought as follows from: ∀α∈[0,1]


$$\frac{a_{1}^{\left(\alpha \right)}-a_{1}}{a_{2}-a_{1}}=\alpha ,\frac{a_{3}-a_{3}^{\left(\alpha \right)}}{a_{3}-a_{2}}=\alpha$$

We bring:

$$a_{1}^{\left(\alpha \right)}=(a_{2}-a_{1})\alpha +a_{1}$$

$$a_{3}^{\left(\alpha \right)}=(a_{3}-a_{2})\alpha +a_{3}$$

Thus:

$$A_{\alpha }=[a_{1}^{\left(\alpha \right)},a_{3}^{\left(\alpha \right)}]=[(a_{2}-a_{1})\alpha +a_{1},-(a_{3}-a_{2})\alpha +a_{3}]$$

Additionally, this method has demonstrated that various uncertain scenarios modeled using Triangular Fuzzy Numbers (TFN) yield superior results in addressing Multi-Criteria Decision-Making (MCDM) problems (Rajak and Shaw, 2019). Options are evaluated based on their proximity to the ideal solutions, with the final selection determined by the resulting rankings. The primary objective of the Fuzzy TOPSIS method aligns with that of the standard TOPSIS method within a fuzzy context: identifying the best combination of variables to achieve the optimal value for the target variable.

## 4 Results and discussion

This section is organized into subheadings and concisely and precisely describes the experimental results, their interpretation, and the conclusions drawn from the experiments.

### 4.1 Results of the application of questionnaires to mezcaleros

Most *mezcaleros* interviewed used an industrial process, and only three had an ancestral process for producing *mezcal*. Most also make part of their *mezcal* for consumption or festivities. Similarly, 60% of the interviewees do not have their *mezcal* brand, while the rest have a brand, and their *mezcal* was ancestral in one case, artisanal in two cases, and industrial in one case. Female participation in the process arises in sales; most interviewees stated that their wives or daughters oversaw sales, and the rest paid staff to do so or did so themselves.

Nonaka and Takeuchi’s four forms of knowledge conversion were also identified in the elaboration of *mezcal* (Nonaka et al., 2000; Nonaka and Teece, 2001). These were socialization, where experiences and technical skills were shared; externalization, which refers to when knowledge becomes explicit; combination, when new knowledge is formed from the union of explicit knowledge; and internalization, when explicit knowledge becomes tacit, when *mezcaleros* learn by doing (Nonaka and Teece, 2001). The following details are how each of these processes was identified.

*Socialization* could be identified when the *mezcaleros* make *tequio*, a collaborative process where community members support each other for a specific purpose. Because the *mezcaleros* have few workers for their operation, between one and two, in the processes that need more personnel, they ask for help from other *mezcaleros* in their community or relatives to support them with their process, being the reciprocal help. When those who helped require labor for their process, those who initially asked for help cooperate in the process of those who helped them. They call this process *tequio*, a way in which they inadvertently share their knowledge through support among the community members without needing a teaching process. This knowledge is shared by experience.

According to Álvarez Hernández and Mercado Salgado (2022), the interconnection between producers is latent in collaborative activities through links of reciprocity as channels to share and exchange resources (tangible and intangible) in an everyday and informal manner (Mohtar et al., 2019). There is a connection between the producers; for example, they maintain the supply of seedlings and pineapple. They share organic fertilizers and insecticides for the cultivation of maguey; instruments are provided for the production of *mezcal*, and the production facilities (alembic) are even shared, sometimes under the halfway deal, that is, the *agave* producer delivers the ripe pineapples to the *mezcal* master and the *mezcal* obtained is distributed between both in equal parts. Among them, there is interest in defending the traditional production process, and there is the space where their customs live: when there is an *agave* harvest and *mezcal* production, the producers support and benefit each other by avoiding paying labor (Mohtar et al., 2019).

Some *mezcaleros* consciously share their knowledge with their descendants and, in a few cases, with their wives. In turn, the knowledge was transmitted to the *mezcaleros* by their ancestors. In this way, knowledge is inherited and transmitted between generations. When they do not have close descendants as their children, they sometimes share it with other relatives, but this conscious transfer is not made to people outside their families. Generally, the conscious transmission process occurs among men, but in some cases, when *mezcaleros* do not have children, they transmit it to their daughters or wives. With this, it is patent that individuals create knowledge, so it cannot be made without them; that is, it is born at the personal level but unites, in turn, to become a collective process (Luque and Rodríguez, 2019). Socialization with external actors such as universities and the government are almost nil since very few producers receive any support for it or have networks with these entities. The government has supported only two of those interviewed in planting *agave*.

The *externalization* of knowledge could be perceived when they modify their recipes for the market’s needs. For example, some make special orders by adding fruits or herbs. Others have gone from an artisanal process to an industrialized one to increase their production volume, from not being certified to getting certified. In contrast, most interviewees keep their knowledge in their memory without a physical record.

The *combination* could be identified when some *mezcaleros* combine ancestral or artisan knowledge with other processes requiring technology. For example, in cooking, some use equipment such as copper stills, and when they verify their quality, they carry out laboratory tests. In contrast, others use clay pots in their cooking and social networks in their sale. With the above, the transfer of knowledge and values of ancestral knowledge, transmitted from generation to generation, is evidenced (Olivares, 2014). Likewise, significant learning of this ancestral knowledge is highlighted (Flores Torres et al., 2021; Food and Agriculture Organization of the United Nations, 2010).

The *mezcaleros* who have gone through a certification process and changed to a more industrialized process allow the *internalization* of knowledge as they accept suggestions and document their process with written records or saved *mezcal* from previous years’ productions. In addition, those who have an industrialized process or who have younger generations involved in the process are those who have documented their knowledge, which facilitates the transmission of explicit knowledge.

The above has been captured in a Bayes Network. This model describes the relationships between the variables described by the experts.

### 4.2 Results of proposed model

**Figure 4:**
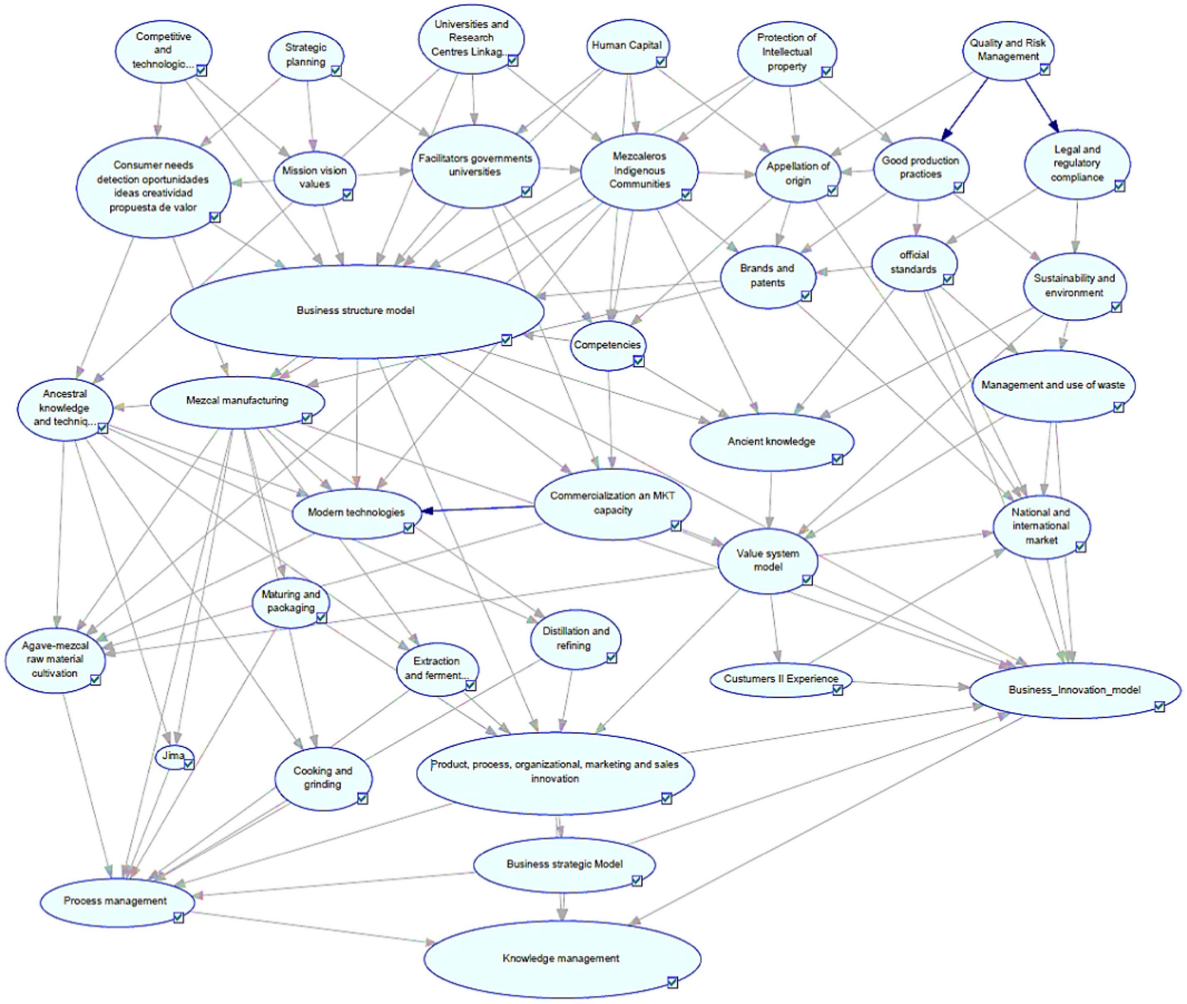
Bayes network configured for *Mezcal* scientific and ancestral knowledge management.

The specialists’ expertise was captured in a Bayesian Network, which vividly illustrates the intricate relationships among the variables that constitute Knowledge Management in Mezcal production. This network serves as a visual and analytical tool, highlighting how different factors interact and influence each other within the production process. The arcs connecting the nodes represent the interdependencies between these variables, as shown in Figure 4. These connections are crucial for understanding how changes in one variable can impact others, affecting the overall system.

In total, 37 nodes were defined, each representing a specific variable within the Knowledge Management framework. Six parent nodes were identified, which serve as primary influencers within the network. The remaining nodes are dependent on these parent nodes, creating a complex web of 191 arcs that map out their relationships and dependencies.

Each node was modeled with three possible states: optimal, regular, or deficient. This modeling approach allows for a detailed analysis of the system’s performance under various conditions, providing insights into potential areas for improvement and optimization. By examining the states of these nodes, stakeholders can identify which aspects of the Knowledge Management process are functioning well and which may require attention or intervention.

Overall, this Bayesian Network offers a comprehensive and dynamic view of the Knowledge Management system in Mezcal production. It enables a deeper understanding of its complexities and facilitates more informed decision-making.

The probability values for each variable’s states define the strength of the relationships represented by the arcs in the Bayesian Network. These arcs illustrate how changes in one variable can influence others within the network. After configuring the Bayesian Network based on expert knowledge, we assess the probability of achieving optimal, regular, and deficient outcomes for the target variable, Knowledge Management.

The Bayesian Network configuration involves setting the initial conditions and dependencies among variables, which domain experts inform. This setup allows us to simulate various scenarios and predict the likelihood of different outcomes. The analysis reveals that, with the current configuration, there is an 85% probability of achieving optimal results for Knowledge Management. This high probability indicates a strong positive influence of the configured variables on the target outcome.

**Figure 5:**
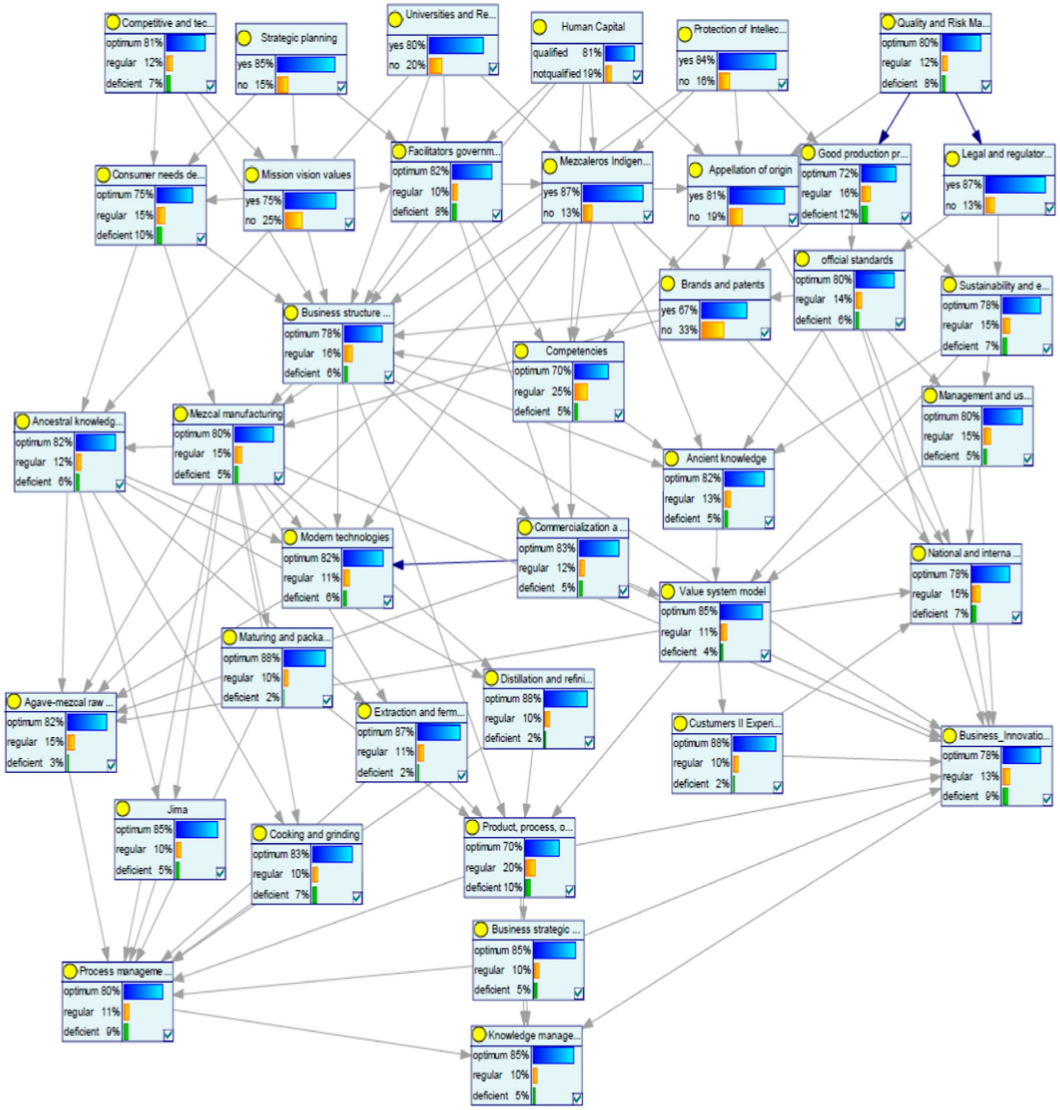
Evaluation of probabilities for Bayes network model.

Figure 5 visually represents these findings, showing the network of variables and their interconnections. The arcs’ thickness and directionality highlight the strength and nature of the relationships, providing a clear understanding of how each variable contributes to the overall probability of achieving optimal Knowledge Management.

Next, the most relevant variables for classification were identified using the Information Gain metric (Table 1). This metric measures the expected amount of information a variable contributes to the classification process, helping to pinpoint which variables are most informative. The analysis revealed that the variables most closely associated with the traditional knowledge scenario in the Mezcal production process include Management and Use of Waste, scientific and ancestral knowledge and Techniques, Denomination of Origin, Jima, and Mezcaleros Indigenous Communities.

**Table 1:** Variables with higher values of the information gain metric.

Variable	Information gain value
Management and use of waste	0.69
Scientific and ancestral knowledge	0.59
Denomination of origin	0.30
Jima	0.33
Mezcaleros indigenous communities	0.29

Management and Use of Waste refers to the efficient handling and repurposing of by-products generated during Mezcal production, which is crucial for sustainable practices. Scientific and Ancestral Knowledge and Techniques encompass the blend of modern scientific methods and traditional practices passed down through generations, ensuring the authenticity and quality of Mezcal. The Denomination of Origin is a certification that denotes the geographical origin of Mezcal, guaranteeing its unique characteristics and quality. Jima is the process of harvesting agave plants, a critical step in Mezcal production. Mezcaleros Indigenous Communities are the local producers and artisans with deep-rooted knowledge and skills in Mezcal making.

These variables were identified as the most relevant and used as a reference for the Fuzzy TOPSIS method. The Fuzzy TOPSIS method is a multi-criteria decision-making approach that evaluates and ranks alternatives based on their performance across multiple criteria. By incorporating these key variables, the method aims to comprehensively and accurately assess the Mezcal production process, ensuring that the most critical factors are considered.

The TOPSIS Fuzzy Method includes six steps: I. Generate a decision matrix, II. Construct the normalized decision matrix, III. Generate the weighted normalized decision matrix, IV. Determine the fuzzy positive ideal solution (FPIS, A*) and the fuzzy negative ideal solution $(\text{FNIS},A^{-}),$ V. Compute the distance between each option and the fuzzy positive ideal solution A* and the distance between each option and the fuzzy negative ideal solution $A^{-},$ VII. Compute the closeness coefficient and classify the choices.

#### 4.2.1 Step 1: create a decision matrix

This study evaluates four criteria and ranks five options using the Fuzzy TOPSIS method. The table below outlines the type of each criterion and the corresponding weight assigned by experts in the field based on their experience and knowledge (Table 2).

**Table 2:** Characteristics of criteria.

S. no.	Name	Type	Weight
1	Management and use of waste	+	(0.050, 0.150, 0.800)
2	Scientific and ancestral knowledge	+	(0.060, 0.120, 0.820)
3	Denomination of origin	+	(0.090, 0.190, 0.810)
4	*Jima*	+	(0.050, 0.100, 0.850)
5	*Mezcaleros* indigenous communities	+	(0.080, 0.130, 0.890)

The Table 3 below presents the fuzzy scale utilized in the model.

**Table 3:** Fuzzy scale.

Code	Linguistic terms	L	M	U
1	Optimum	0.2	0.5	0.81
2	Regular	0.09	0.1	0.12
3	Deficient	0.03	0.05	0.07

The alternatives are assessed using a variety of criteria, and the results are presented in the decision matrix below. If multiple experts are involved in the evaluation, the matrix represents the arithmetic mean of their assessments (see Table 4).

**Table 4:** Decision matrix.

Alternative#	Management and use of waste	Scientific and ancestral knowledge	Denomination of origin	*Jima*	Mezcaleros indigenous communities
Alternative 1	(0.040, 0.058, 0.078)	(0.030, 0.050, 0.070)	(0.117, 0.225, 0.342)	(0.060, 0.075, 0.095)	(0.098, 0.158, 0.227)
Alternative 2	(0.040, 0.058, 0.078)	(0.088, 0.150, 0.218)	(0.145, 0.300, 0.465)	(0.040, 0.058, 0.078)	(0.108, 0.167, 0.235)
Alternative 3	(0.050, 0.067, 0.087)	(0.030, 0.050, 0.070)	(0.080, 0.092, 0.112)	(0.068, 0.133, 0.202)	(0.108, 0.167, 0.235)
Alternative 4	(0.060, 0.075, 0.095)	(0.040, 0.058, 0.078)	(0.098, 0.158, 0.227)	(0.040, 0.058, 0.078)	(0.108, 0.167, 0.235)
Alternative 5	(0.068, 0.133, 0.202)	(0.050, 0.067, 0.087)	(0.108, 0.167, 0.235)	(0.030, 0.050, 0.070)	(0.108, 0.167, 0.235)

#### 4.2.2 Step 2: create the normalized decision matrix

Based on the positive and negative ideal solutions, a normalized decision matrix can be computed by the subsequent equations:

$${\tilde{r}}_{\mathrm{ij}}=(\frac{a_{\mathrm{ij}}}{c_{j}^{*}},\frac{b_{\mathrm{ij}}}{c_{j}^{*}},\frac{c_{\mathrm{ij}}}{c_{j}^{*}});c_{j}^{*}=\max_{i}\;c_{\mathrm{ij}};\text{Positive ideal solution}$$

$${\tilde{r}}_{\mathrm{ij}}=(\frac{a_{j}^{-}}{c_{\mathrm{ij}}},\frac{a_{j}^{-}}{b_{\mathrm{ij}}},\frac{a_{j}^{-}}{a_{\mathrm{ij}}});a_{j}^{-}=\min_{i}\;a_{\mathrm{ij}};\text{Negative ideal solution}$$

The normalized decision matrix is exposed in the Table 5 below.

**Table 5:** A normalized decision matrix.

Alternative #	Management and use of waste	Scientific ancestral knowledge	Denomination of origin	*Jima*	*Mezcaleros* indigenous communities
Alternative 1	(0.198, 0.287, 0.386)	(0.138, 0.229, 0.321)	(0.252, 0.484, 0.735)	(0.297, 0.371, 0.470)	(0.417, 0.672, 0.966)
Alternative 2	(0.198, 0.287, 0.386)	(0.404, 0.688, 1.000)	(0.312, 0.645, 1.000)	(0.198, 0.287, 0.386)	(0.460, 0.711, 1.000)
Alternative 3	(0.248, 0.332, 0.431)	(0.138, 0.229, 0.321)	(0.172, 0.198, 0.241)	(0.337, 0.658, 1.000)	(0.460, 0.711, 1.000)
Alternative 4	(0.297, 0.371, 0.470)	(0.183, 0.266, 0.358)	(0.211, 0.340, 0.488)	(0.198, 0.287, 0.386)	(0.460, 0.711, 1.000)
Alternative 5	(0.337, 0.658, 1.000)	(0.229, 0.307, 0.399)	(0.232, 0.359, 0.505)	(0.149, 0.248, 0.347)	(0.460, 0.711, 1.000)

#### 4.2.3 Step 3: create the weighted normalized decision matrix

Considering the different weights of each criterion, the weighted normalized decision matrix can be calculated by multiplying the weight of each criterion by the corresponding value in the normalized fuzzy decision matrix according to the following formula:

$${\tilde{v}}_{\mathrm{ij}}={\tilde{r}}_{\mathrm{ij}}.{\tilde{w}}_{\mathrm{ij}}$$

Where ${\tilde{w}}_{\mathrm{ij}}$ represents weight of criterion $c_{j.}$

The following Table 6 presents the weighted normalized decision matrix

**Table 6:** The weighted normalized decision matrix.

Alternative #	Management and use of waste	Scientific and ancestral knowledge	Denomination of origin	*Jima*	*Mezcaleros* indigenous communities
Alternative 1	(0.010, 0.043, 0.309)	(0.008, 0.028, 0.263)	(0.023, 0.092, 0.596)	(0.015, 0.037, 0.400)	(0.033, 0.087, 0.860)
Alternative 2	(0.010, 0.043, 0.309)	(0.024, 0.083, 0.820)	(0.028, 0.123, 0.810)	(0.010, 0.029, 0.328)	(0.037, 0.092, 0.890)
Alternative 3	(0.012, 0.050, 0.345)	(0.008, 0.028, 0.263)	(0.015, 0.038, 0.195)	(0.017, 0.066, 0.850)	(0.037, 0.092, 0.890)
Alternative 4	(0.015, 0.056, 0.376)	(0.011, 0.032, 0.293)	(0.019, 0.065, 0.395)	(0.010, 0.029, 0.328)	(0.037, 0.092, 0.890)
Alternative 5	(0.017, 0.099, 0.800)	(0.014, 0.037, 0.327)	(0.021, 0.068, 0.409)	(0.007, 0.025, 0.295)	(0.037, 0.092, 0.890)

#### 4.2.4 Step 4: find the fuzzy positive ideal solution (FPIS, a*) and the fuzzy negative ideal solution ( $\text{FNIS},A^{-}$ )

The FPIS and FNIS of the alternatives can be expressed on this way:

$$A^{*}=\{{\tilde{v}}_{1}^{*},{\tilde{v}}_{2}^{*},\ldots ,{\tilde{v}}_{n}^{*}\}=\{(\max_{j}v_{\mathrm{ij}}|i\in B),(\min_{j}v_{\mathrm{ij}}|i\in C)\}$$

$$A^{-}=\{{\tilde{v}}_{1}^{-},{\tilde{v}}_{2}^{-},\ldots ,{\tilde{v}}_{n}^{-}\}=\{(\min_{j}v_{\mathrm{ij}}|i\in B),(\max_{j}v_{\mathrm{ij}}|i\in C)\}$$

Where ${\tilde{v}}_{i}^{*}\;$ is the maximum value of i for all the alternatives, and ${\tilde{v}}_{1}^{-}$ is the minimum value of i for all the alternatives. *B* and *C* embody the positive and negative ideal solutions, respectively. The positive and negative ideal solutions are exposed in the Table 7 below.

**Table 7:** The positive and negative ideal solutions.

Factor	Positive ideal	Negative ideal
Management and use of waste	(0.017, 0.099, 0.800)	(0.010, 0.043, 0.309)
Scientific and ancestral knowledge	(0.024, 0.083, 0.820)	(0.008, 0.028, 0.263)
Denomination of origin	(0.028, 0.123, 0.810)	(0.015, 0.038, 0.195)
*Jima*	(0.017, 0.066, 0.850)	(0.007, 0.025, 0.295)
*Mezcaleros* indigenous communities	(0.037, 0.092, 0.890)	(0.033, 0.087, 0.860)

#### 4.2.5 Step 5: compute the distance between each alternative and the fuzzy positive ideal solution $A^{*}$ and the distance between each alternative and the fuzzy negative ideal solution $A^{-}$

The distance between the alternatives and FPIS and the distance amid the alternatives and FNIS are correspondingly calculated as follows:

$$S_{i}^{*}=\sum_{j=1}^{n}d({\tilde{v}}_{\mathrm{ij}},{\tilde{v}}_{j}^{*})\;i=1,2,\ldots ,m$$

$$S_{i}^{-}=\sum_{j=1}^{n}d({\tilde{v}}_{\mathrm{ij}},{\tilde{v}}_{j}^{-})\;i=1,2,\ldots ,m$$

d is the distance between two fuzzy numbers, when given two triangular fuzzy numbers ( $a_{1},b_{1},c_{1}$ ) and ( $a_{2},b_{2},c_{2}$ ), the distance between the two can be in the following way:

$$d_{v}({\tilde{M}}_{1},{\tilde{M}}_{2})=\sqrt{\frac{1}{3}[{\left(a_{1}-a_{2}\right)}^{2}+{\left(b_{1}-b_{2}\right)}^{2}+{\left(c_{1}-c_{2}\right)}^{2}]}$$

Note that $d({\tilde{v}}_{\mathrm{ij}},{\tilde{v}}_{j}^{*})$ and $d({\tilde{v}}_{\mathrm{ij}},{\tilde{v}}_{j}^{-})$ are crisp numbers.

Table 8 expresses the distance between positive and negative ideal solutions.

**Table 8:** Distance from positive and negative ideal solutions.

Alternative	Distance from the positive ideal	Distance from the negative ideal
Alternative 1	1.012	0.295
Alternative 2	0.587	0.719
Alternative 3	0.946	0.36
Alternative 4	1.095	0.211
Alternative 5	0.841	0.466

#### 4.2.6 Step 6: calculate the closeness coefficient and rank the alternatives

The closeness coefficient of each alternative is computed in this way:

$${\mathrm{CC}}_{i}=\frac{S_{i}^{-}}{S_{i}^{+}+S_{i}^{-}}$$

**Figure 6:**
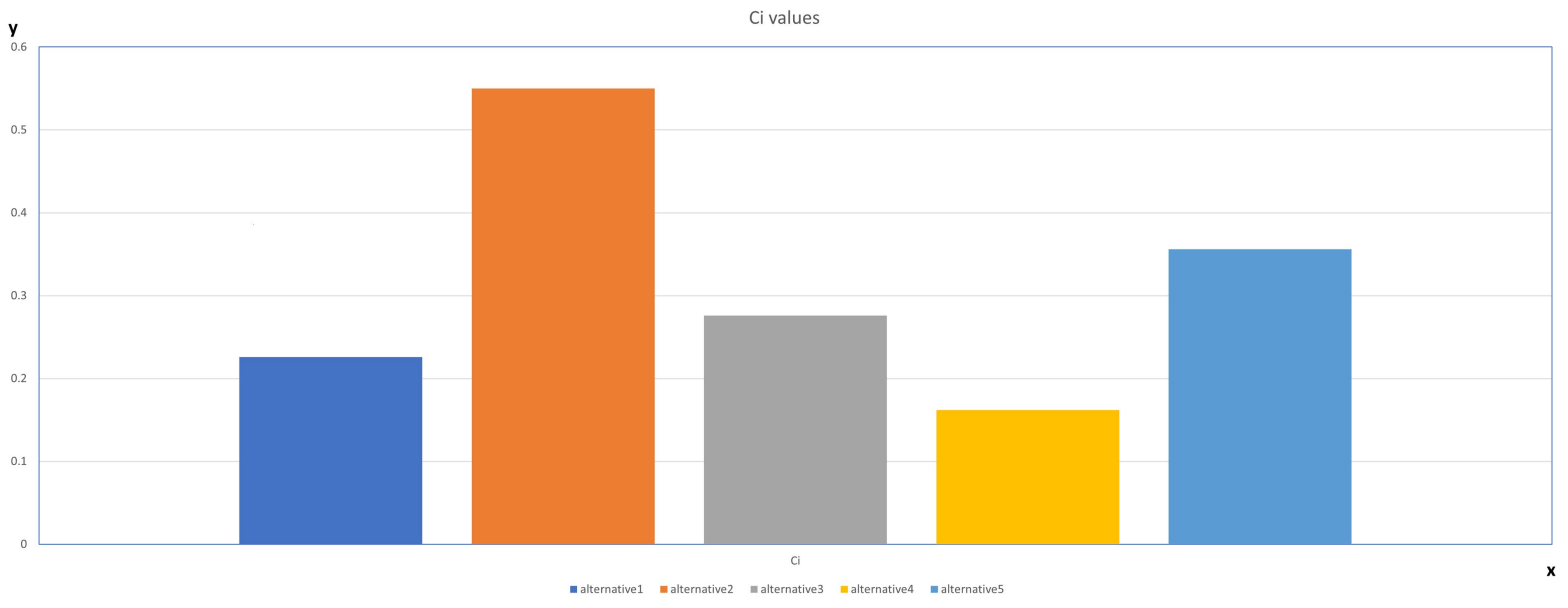
Based on the closeness coefficient, alternative 2 is the best combination of variables to reach knowledge management.

The best alternative is closest to the FPIS and farthest from the FNIS. The table below displays the alternatives’ closeness coefficients and rankings. The C values show that the best combination of variables is the one indicated by Alternative 2, which corresponds to first place in the ranking (Table 9 and Figure 6).

**Table 9:** Closeness coefficient.

Alternative	Ci	Rank
Alternative 1	0.226	4
**Alternative 2**	***0.55***	*1*
Alternative 3	0.276	3
Alternative 4	0.162	5
Alternative 5	0.356	2

The values ​​marked in bold show that the best combination of variables is the one indicated by Alternative 2.

### 4.3 The best alternative explanation (alternative 2)

**Figure 7:**
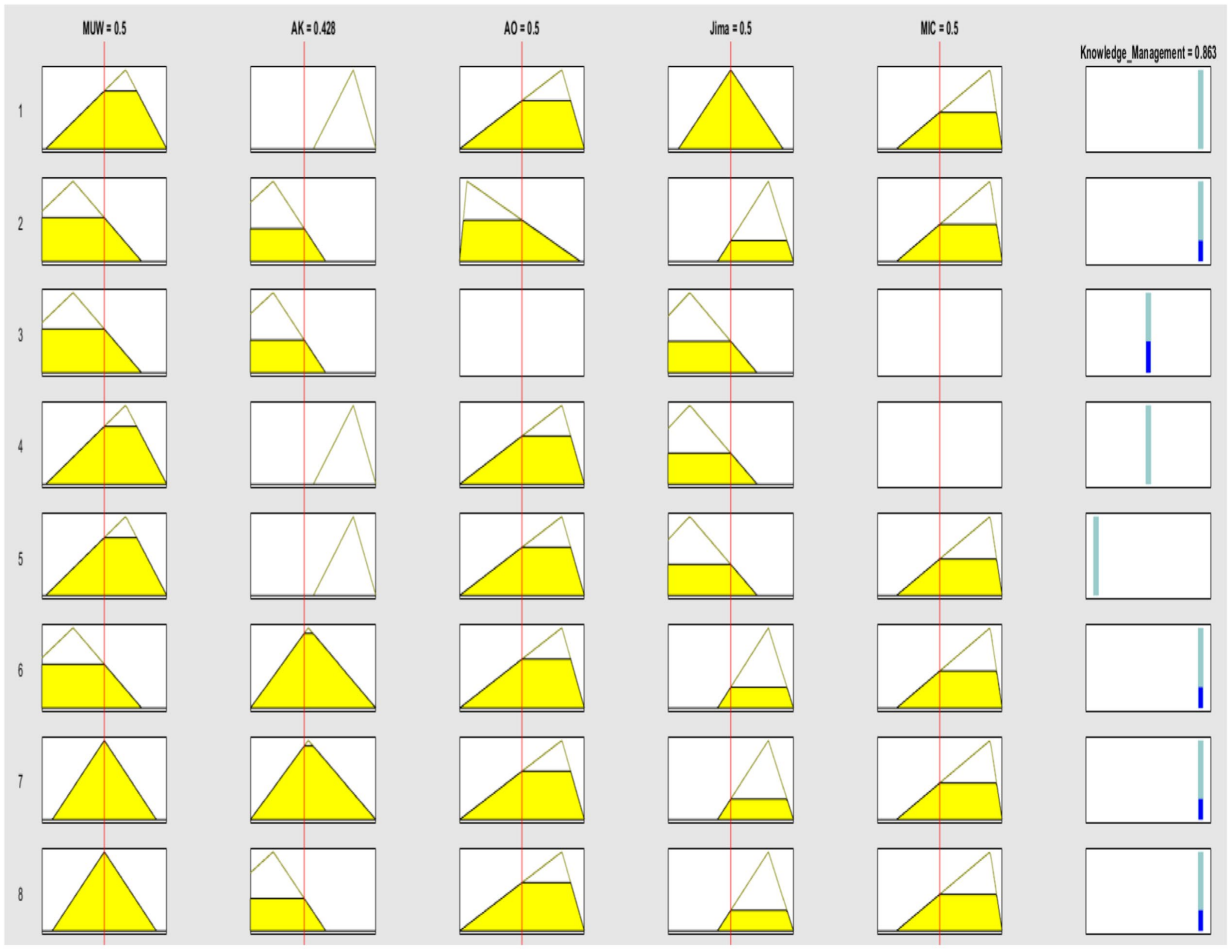
Graphical rules developed from the data patterns.

A surface graphic illustrates the relationship among the component variables for Alternative 2, as computed by the Fuzzy TOPSIS model. This relationship is explained by the rules derived from the patterns generated in the dataset (Figure 7). Each column represents a variable: Management Use of Waste (MUW), Scientific and Ancestral Knowledge (AK), Denomination of Origin (AO), and Jima, with Knowledge Management (KM) as the target variable. The yellow area indicates the value for each variable, with the first four columns showing the values for each variable and the fifth column displaying the value reached by the target variable. The rows represent the rules generated by the fuzzy model.

In this work, we developed several graphical representations of the possible combinations of the variables considered, which have been referred to as graphical rules (Figure 7). These are based on the numerical values shown in Table 4, which lists the fuzzy value of each variable and the possible combinations between the variables of interest. This graphical representation shows that the first, sixth, seventh, and eighth rules, corresponding to Alternative 1, offer the best value for the target variable, Knowledge Management.

The graphical rule for Alternative 2 delineates the optimal solutions for combining the five variables under study. Each variable is systematically represented in a column, with its respective values detailed in each row, consistently arranged in a triangular format. This structured approach allows for a clear and organized presentation of data.

In this format, the last column is particularly significant as it displays the value achieved by the target variable. This value is derived based on the specific combination of values shown in the corresponding row for the other variables. Figure 7 visually encapsulates this relationship, providing a comprehensive overview of how different combinations of the five variables influence the target variable.

By examining these rules, one can discern the most effective combinations of the variables to achieve the desired outcome. This methodical representation highlights the optimal solutions and facilitates a deeper understanding of the interplay between the variables. It underscores the importance of each variable’s contribution to the overall objective, offering valuable insights for decision-making and strategic planning.

**Figure 8:**
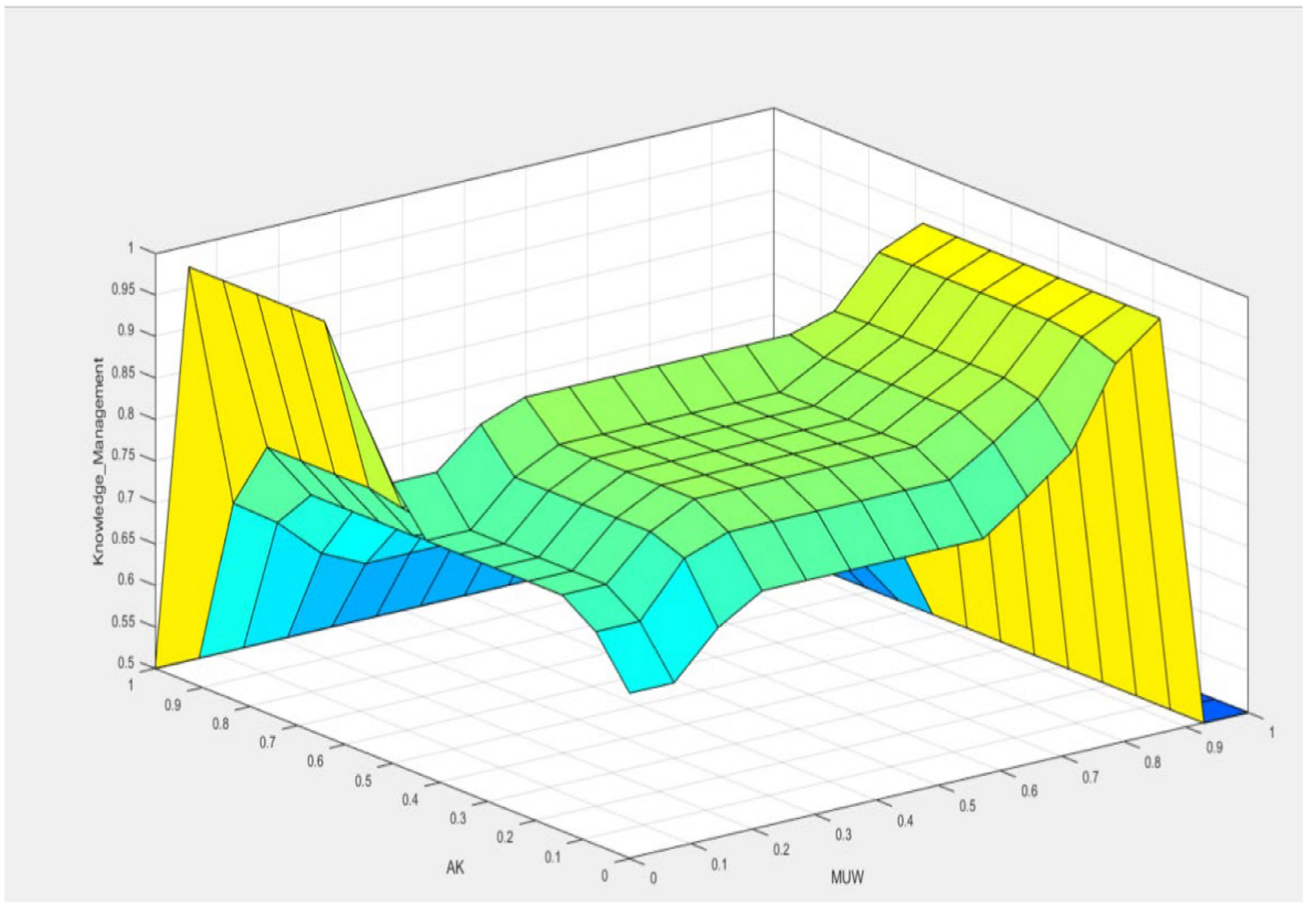
Relationship among the variables knowledge management and management use of waste.

Figure 8 presents the ideal solution identified by the model to elucidate the interplay of variables. This solution suggests that the optimal scenario is achieved when the objective variable (KM) attains its maximum value. That optimal condition is met even if the variables Jima, Management, and Use of Waste are below 0.4. This is conditional on the variables Scientific and Ancestral Knowledge, Denomination of Origin, and Mezcaleros Indigenous Communities having values close to 1.0.

In more detail, the model indicates that the highest value of KM can be reached despite lower values in *Jima*, Management, and Use of Waste. The high values in Scientific and Ancestral Knowledge, Denomination of Origin, and Mezcaleros Indigenous Communities significantly contribute to the overall objective. When near their maximum values, these variables create a favorable environment that compensates for the lower values of the other variables.

**Figure 9:**
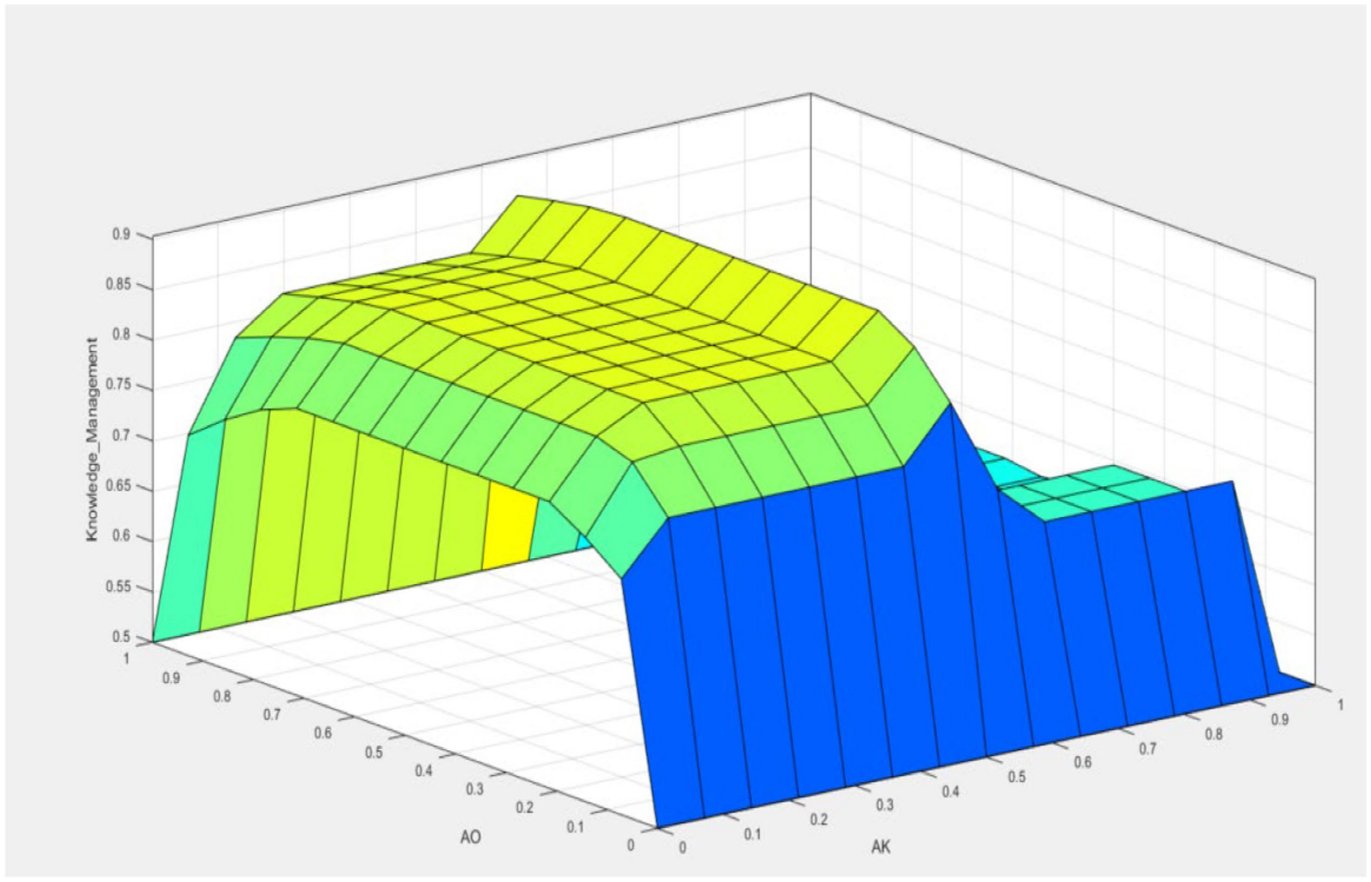
Relationship among the variables denomination of origin, ancestral knowledge, and knowledge management.

Figure 9 further illustrates this relationship by showing how the high values of Scientific and Ancestral Knowledge, Denomination of Origin, and Mezcaleros Indigenous Communities interact to support the objective variable KM. It highlights these variables’ critical role in achieving the optimal solution, demonstrating their importance in the model’s framework.

The surface graphic in Figure 8 vividly illustrates that the target variable, Knowledge Management (KM), achieves its optimal value when the variable Ancestral Knowledge (AK) is at 0.5, and the variable Denomination of Origin (AO) is close to 1. This graphical representation provides a clear visual understanding of how these specific values of AK and AO contribute to maximizing KM.

In summary, the foundation of this analysis was built upon the expert opinions, which were instrumental in developing a Bayesian Network model. This model was constructed by first generating a dataset that captured the patterns and relationships among the variables. Through this process, the relevance of each variable was meticulously determined based on its influence on the classification outcomes.

Once the relevance of the variables was established, their values were strategically combined to identify the optimal combination that would enhance the Knowledge Management value. This approach highlighted the critical variables and provided insights into how their specific values interact to achieve the desired outcome. The model thus serves as a robust tool for understanding and optimizing the factors that contribute to effective Knowledge Management.

## 5 Conclusion

The Bayesian Network model indicates the values for the precedent variables to obtain an 85% probability of raising the optimum state for Knowledge Management, where the most relevant variables Management and use of waste, Ancestral Knowledge, Denomination of Origin, Jima and Mezcaleros_Indigenous_Communities having the probabilities value 80, 82, 85, and 87%, respectively.

Next, these variables were analyzed using the fuzzy TOPSIS technique to search for a better combination of variables and their values to obtain a better result for the Knowledge Management variable, obtaining the positive ideal solutions for combining the five variables studied. The ideal solution shows that the optimal case occurs if the objective variable Knowledge Management reaches its highest value if the Denomination of Origin and Mezcaleros Indigenous Communities have the weights 0.309, 0.820, 0.810, 0.328, and 0.890, respectively, for the highest values for the state Optimum in the fuzzy scale. That denotes the importance of the variables related to the knowledge transmitted from generation to generation with scientific knowledge. The picture’s explanations aim to increase the models’ explicability. Therefore, the values referring to traditional knowledge indicate that preserving and promoting the values generated through them increases knowledge management.

These variables underscore the importance of a model connecting sustainability and preserving cultural and territorial heritage through the Denomination of Origin. That is crucial as it ensures that ancestral knowledge and the management of mezcal knowledge by communities are not overlooked. Despite the ongoing debate about the Denomination of Origin potentially sidelining community practices, traditions, and customs due to standardized processes, these results allow us to view these variables as interconnected networks that facilitate optimal knowledge management.

Mezcal production is a cultural activity and a demonstration of ancestral knowledge that forms the legacy of generations of mezcal masters. This knowledge imparts unique smells and flavors to mezcal, making it an economically viable livelihood when all production processes are utilized comprehensively, and the right marketing channels are explored (Terán-Bustamante et al., 2025).

Thus, in this scenario, one of the main challenges’ producers face is being linked to a production and trade chain. However, this action would require raw materials, equipment, technology, economic resources, assistance, and programs that contribute to a considerable production positioning and adherence to the stipulated standards that allow for promoting access to the Denomination of Origin area.

The linking of these actors is fundamental in social exchange to generate mutual agreements (Molm, 1994). These agreements involve actions that lead to exchanging ideas, vision, knowledge, and experiences and, therefore, generating knowledge that results in cooperation to achieve the expected results (Mubarak et al., 2024).

The management of the scientific and ancestral knowledge model provides insight into the most critical factors that decision-makers and public policy-makers must consider to integrate and connect diverse stakeholders to achieve a higher quality of life for Indigenous and rural communities.

According to Echavarría-Heras et al. (2023), ancestral-traditional knowledge should be considered alongside scientific approaches to gain a better understanding of species interactions. Integrating it provides a better understanding of ecosystem dynamics and the impacts of human activities on the environment. Furthermore, better solutions can be built. By integrating ancestral knowledge, it would be possible to develop better options for establishing conservation and resource management programs (Echavarría-Heras et al., 2023).

This analysis technique can be a reference for future research that seeks to better understand knowledge management in industrial and artisanal processes. For this linkage model to be successful, the main actors—the government and academia—universities and research centers—along with scientific knowledge play an essential role.

## Data Availability

The raw data supporting the conclusions of this article will be made available by the authors, without undue reservation.
